# Hairy/enhancer of Split Homologue-1 Suppresses Vascular Endothelial Growth Factor-induced Angiogenesis via Downregulation of Osteopontin Expression

**DOI:** 10.1038/s41598-017-01018-6

**Published:** 2017-04-18

**Authors:** Xing-Xing Yao, Jing-Bo Lu, Zhi-Dong Ye, Lei Zheng, Qian Wang, Zhi-Qi Lin, Hao Liu, Heng Wan, Fang-Yong Fu, Xian-Ying Huang, Jian-Chen Xiu, Zheng-Jun Liu, Yan-Wei Hu

**Affiliations:** 1 Department of Vascular Surgery, Nanfang Hospital, Southern Medical University, Guangzhou, 510515 China; 2 Laboratory Medicine Center, Nanfang Hospital, Southern Medical University, Guangzhou, 510515 China; 3 Department of Cardio vascular Surgery, China- Japan Friendship Hospital, Beijing, 100029 China; 4 Department of Cardiology, Nanfang Hospital, Southern medical University, Guangzhou, 510515 China

## Abstract

Angiogenesis plays a critical role in the progression and vulnerability of atherosclerotic plaques; however, the orchestration of angiogenesis in atherosclerotic plaque formation remains unclear. The results of microarray analysis, real-time PCR and immunohistochemical analyses showed that Hairy/enhancer of split homologue-1 (Hes-1) expression was significantly decreased, while that of osteopontin (OPN) was increased, in atherosclerotic plaques. Meanwhile, immunofluorescence results demonstrated that both Hes-1 and OPN were expressed in endothelial cells (ECs) of neovessels in atherosclerotic plaques. The results of an *in vitro* study showed that Hes-1 was downregulated, while OPN was upregulated, in a time- and dose-dependent manner in human umbilical vein endothelial cells (HUVECs) by VEGF treatment. In addition, Hes-1 knockdown was found to have transcriptional promotion effect on OPN expression in HUVECs and enhance OPN-induced angiogenesis in response to VEGF. On the contrary, Hes-1 overexpression inhibited OPN expression in HUVECs and reduced angiogenesis *in vitro* and *in vivo*. The results of this study suggest that decreased Hes-1 expression in atherosclerotic plaques exaggerate VEGF-induced angiogenesis by upregulating OPN. Therefore, restoring Hes-1 expression and inhibiting OPN expression may be a promising strategy to prevent vulnerable plaque formation in patients with atherosclerosis.

## Introduction

Atherosclerosis is the principal contributing factor to the pathology of cardiovascular disease^1^. The clinical complications of atherosclerosis are mainly caused by thrombus formation, which result from rupture of a vulnerable atherosclerotic plaque^2^. Importantly, angiogenesis is a key feature of atherosclerosis^3^. The formation of microvessels (angiogenesis) in an atherosclerotic plaque contributes to the development of vulnerable plaques, thereby increasing the risk of rupture^4^. Although the regulation of angiogenesis in atherosclerotic plaques is still under investigation, suppression of angiogenesis is considered a promising therapeutic strategy for the treatment of atherosclerosis.

Hairy/enhancer of split homologue-1 (Hes-1) is a transcriptional repressor of members of the basic helix-loop-helix family of transcription factors. It has been shown that Hes-1 plays a critical role in the regulation of various physiological processes, including cellular differentiation, cycling, apoptosis, and self-renewal^5, 6^. Hes-1-regulated transcription is essential to cardiovascular development and its function has been linked to smooth muscle differentiation, angiogenic processes, arterial-venous cell fate determination, and vascular morphogenesis^7^. In addition, increasing evidence has shown that Hes-1 is involved in the regulation of endothelial cell (EC) proliferation, differentiation, and apoptosis, which are fundamental processes necessary for angiogenesis^8^. Curry *et al*.^9^ demonstrated that Hes-1 expression is regulated in confluent ECs by the JNK signaling pathway, which induces EC growth arrest. However, it remains unknown whether Hes-1 is involved in the progression and vulnerability of atherosclerotic plaques by affecting angiogenesis.

Osteopontin (OPN) is an acidic glycoprotein that is a member of the small integrin-binding N-linked glycoprotein family that was recently found to play both proinflammatory and proatherogenic roles in the pathogenesis of atherosclerotic plaques induced by various atherosclerotic risk factors^10^. Moreover, initial studies have demonstrated that OPN not only promotes arteriosclerosis but is also closely associated with angiogenesis^11, 12^. Wang *et al*.^13^ revealed that OPN directly stimulates angiogenesis via the avβ3/PI3-K/AKT/eNOS/NO signaling pathway and Junaid *et al*.^14^ found that OPN improves survival and differentiation of ECs during angiogenesis by binding to the integrin αvβ3 and triggering downstream signaling. These results indicate that OPN is a promising therapeutic target for angiogenesis in atherosclerosis. Although the mechanism underlying OPN regulation during angiogenesis remains unclear, Matsue *et al*.^15^ showed that Hes-1 overexpression suppressed OPN transcription, suggesting that Hes-1 is a potential regulator of OPN. In atherosclerotic plaques, vascular endothelial growth factor (VEGF) produced by inflammatory cells and/or ECs has been implicated in the initiation of intraplaque angiogenesis and promotion of plaque formation, which eventually results in intraplaque hemorrhage^16^. As a well-studied growth and angiogenic cytokine, VEGF plays a prominent role in normal and pathological angiogenesis.^17^ VEGF has been verified to promote angiogenesis and vascular permeability by activating of the signalling pathways such as ERK/MAPK, PI3K/Akt and the stress kinase p38MAPK, as well as the ERK1/2 and the JAK/STAT pathways^18^. Hes-1-monitored transcription has been shown to be involved in the VEGF signaling pathway^19^. Thomas *et al*.^20^ showed that Hes-1 was a signaling hub in early retinal progenitor cells that integrates downstream VEGF signaling, although the role of Hes-1 in VEGF-induced angiogenesis remains unclear. In addition to Hes-1, increasing studies have found that VEGF induces OPN expression in ECs^21–23^. However, whether Hes-1 plays a role in VEGF-induced angiogenesis in atherosclerotic plaque formation via OPN regulation has not yet been elucidated.

The results of this study demonstrated that Hes-1 was significantly downregulated, while OPN was upregulated in atherosclerotic plaques, and that both Hes-1 and OPN were expressed in ECs of neovessels in atherosclerotic plaques. Moreover, Hes-1 knockdown in human umbilical vein endothelial cells (HUVECs) augmented VEGF-induced upregulation of OPN and promoted angiogenesis, but Hes-1 overexpression suppressed VEGF-induced elevation of OPN and inhibited angiogenesis, suggesting that decreased Hes-1 expression may promote VEGF-angiogenesis by upregulating OPN in atherosclerotic plaques and restoring Hes-1 expression to stabilize vulnerable plaques by suppressing intraplaque angiogenesis. These findings indicate that Hes-1 and OPN are novel modulators of the progression and vulnerability of atherosclerotic plaques, and that dysregulation of these factors may contribute to the pathogenesis of vulnerable atherosclerotic plaques.

## Results

### Down-regulation of Hes-1 in human atherosclerotic plaques

**Figure 1:**
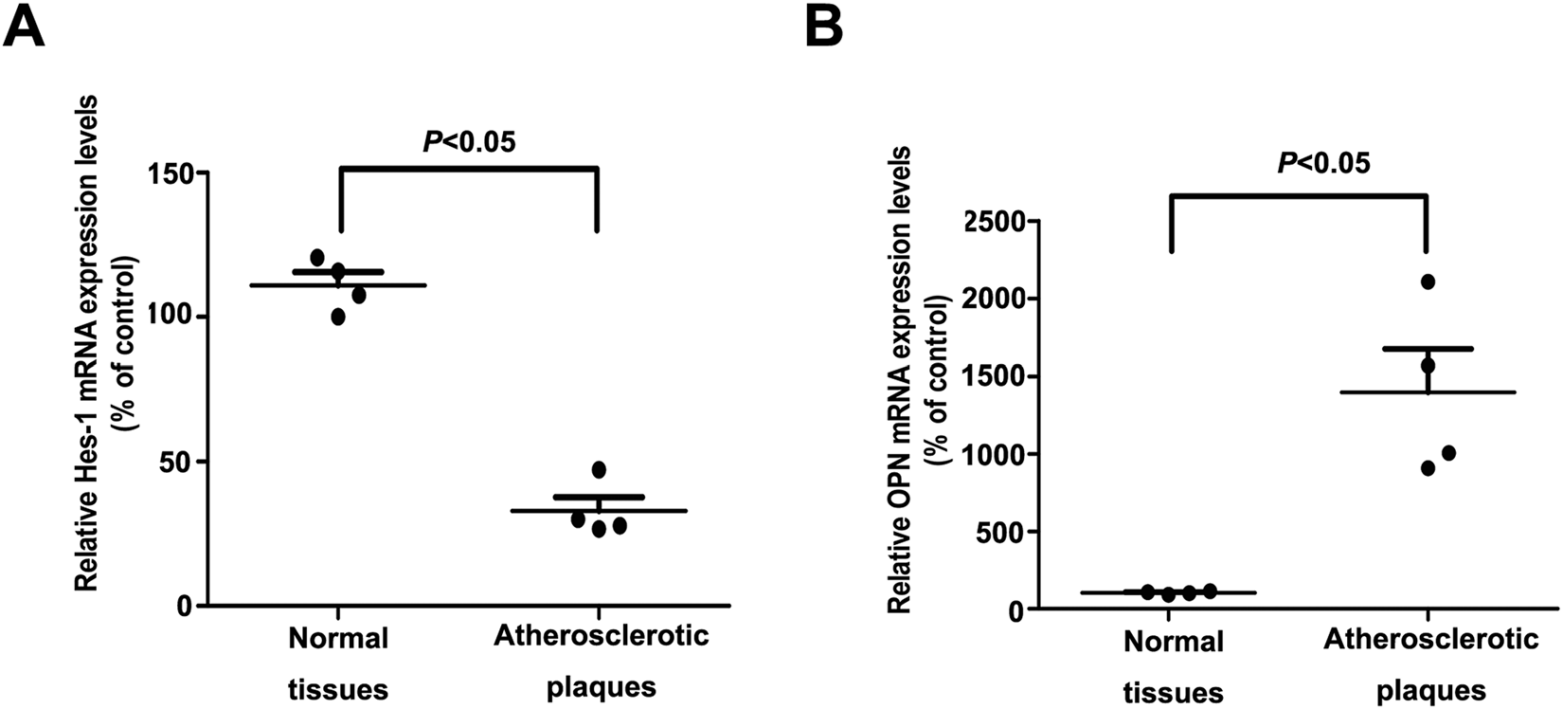
Down-regulation of Hes-1 and up-regulation of OPN in human atherosclerotic plaques. (**A**,**B**) Hes-1 and OPN expression levels in atherosclerotic plaques and normal arterial intima tissues were measured by real-time quantitative PCR. The expression of GAPDH was used as an internal control. Data are presented as the mean ± S.D. of three independent experiments, each performed in triplicate.

**Figure 2:**
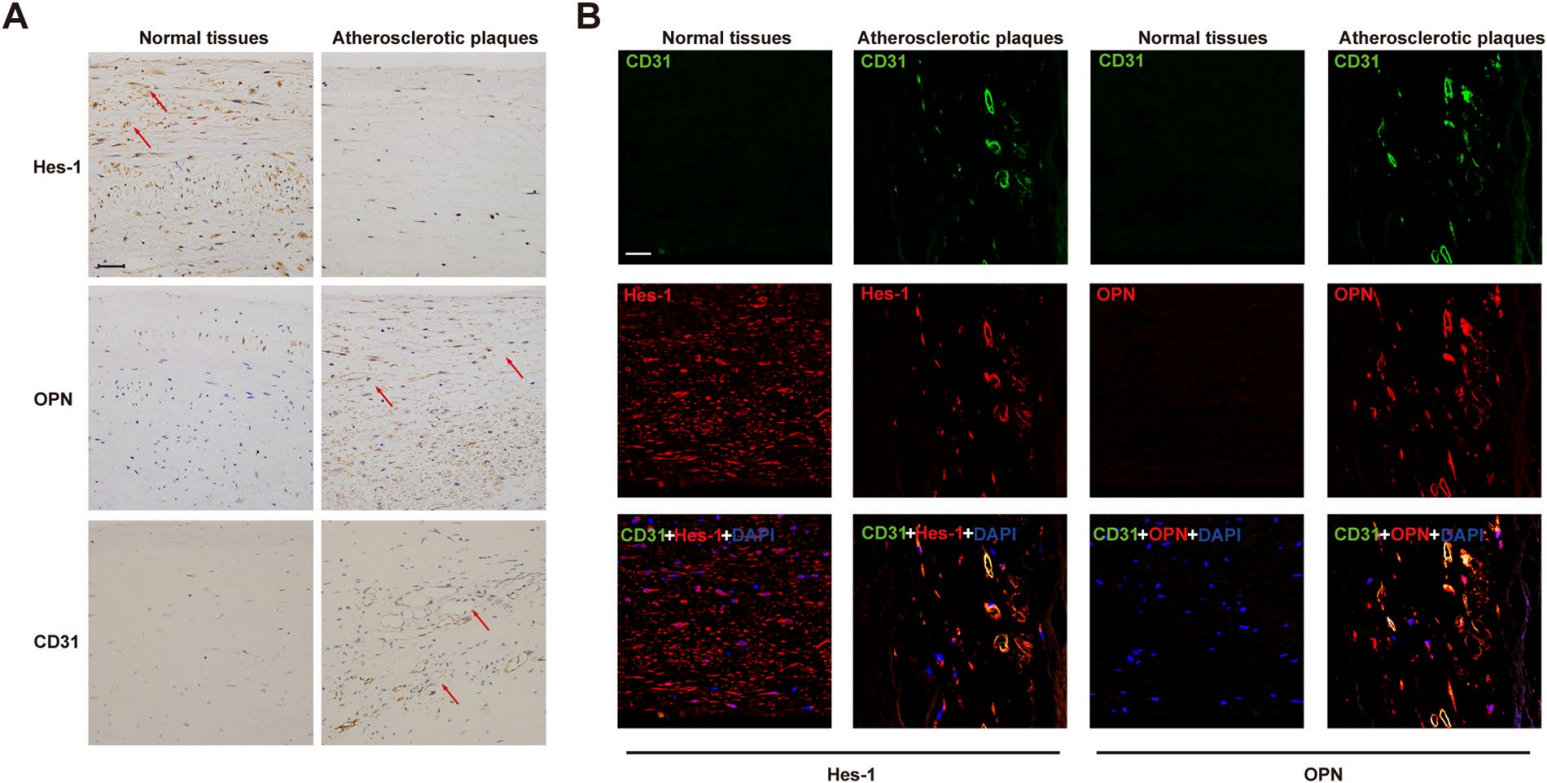
Hes-1 was downregulated in neovessels of atherosclerotic plaques and OPN was upregulated in ECs of neovessels in atherosclerotic plaques. (**A**) Immunohistochemical staining of Hes-1, OPN, and CD31 in normal arterial intima tissues and atherosclerotic plaques. Representative photomicrographs at ×40 magnification of stained tissues sections demonstrating greater staining for Hes-1 and OPN in arterial intima tissues. Bar: 50 μm. (**B**) Colocalization of CD31 and Hes-1 or OPN in normal arterial intima tissues and atherosclerotic plaques. Merged images in which the expression of CD31 is shown in green and the expression of Hes-1 and OPN are shown in red. The yellow fluorescence represents the coexpression of CD31 and Hes-1/ OPN in endothelial cells of neovessels in atherosclerotic plaques. Note that expression was limited to atherosclerotic plaques. Normal arterial intima typically lacked signals of CD31 and neovessels. Nuclei are counterstained with DAPI (blue). Original magnification 40x; Bar: 50 μm.

Hes-1 plays an important role in angiogenesis^8^. However, whether Hes-1 is dyregulated in atherosclerotic plaques and whether this downregulation affects angiogenesis in atherosclerotic plaques remain unknown. To investigate possible changes in RNA expression during atherosclerosis, microarray analysis of atherosclerotic plaques and normal arterial intima tissues was performed (Table 1). The results revealed that Hes-1 was decreased by 20.68-fold (*p* < 0.005) in atherosclerotic plaques. To further investigate the downregulation of Hes-1 in atherosclerotic plaques, expression levels of Hes-1 in four atherosclerotic plaques and four normal arterial intima tissues were determined using real-time PCR, immunohistochemical and immunofluorescence analyses. The results of real-time PCR analysis showed that Hes-1 mRNA levels were significantly lower in atherosclerotic plaques than in normal arterial intima tissues (Fig. 1A). Immunohistochemical staining showed that Hes-1 was expressed in sheet-like patterns within the arterial intima (Fig. 2A). The mean optical density of Hes-1 staining was significantly greater in normal arterial intima, which was consistent with the results of RT-PCR (Table 2). Studies have shown that CD31 is a superior marker for evaluation of human angiogenesis^24^. CD31 / Hes-1 double immunofluorescence labelling further confirmed that Hes-1 was expressed in ECs of neovessels in atherosclerotic plaques. Immunofluorescence results also showed a significant decrease in Hes-1 expression in atherosclerotic plaques, as compared to normal arterial intima (Fig. 2B). These results further demonstrated that Hes-1 was downregulated in neovessels of atherosclerotic plaques and that Hes-1 might be might be involved in intraplaque angiogenesis.

**Table 1:** Demographic data.

	Age	Gender	Tissue	Pathological stage
Case#1	25	Male	Renal artery intima	Normal
Case#2	31	Male	Renal artery intima	Normal
Case#3	19	Male	Renal artery intima	Normal
Case#4	28	Male	Renal artery intima	Normal
Case#5	48	Male	Renal artery atherosclerosis	III
Case#6	55	Male	Renal artery atherosclerosis	III-IV
Case#7	69	Male	Renal artery atherosclerosis	IV
Case#8	57	Male	Renal artery atherosclerosis	V

**Table 2:** Comparison of Hes-1, OPN concentrations in atherosclerotic plaques and normal arterial intimal tissues using Immunohistochemistry, Immunofluorescence and Image Analysis.

	Immunohistochemistry			Immunofluorescence		
Protein	normal tissues	atherosclerotic plaques	*p*	normal tissues	atherosclerotic plaques	*p*
Hes-1	54.89 ± 19.73	4.26 ± 2.14	<0.05	99.95 ± 19.32	9.42 ± 10.15	<0.05
OPN	9.79 ± 7.46	96.75 ± 22.78	<0.05	4.00 ± 3.40	144.1 ± 13.97	<0.05

Figure presented are mean optical densities minus background staining from negative slides ± standard error. Hes-1, hairy/enhancer of split homologue-1; OPN indicates osteopontin.

### VEGF downregulated Hes-1 expression in HUVECs

**Figure 3:**
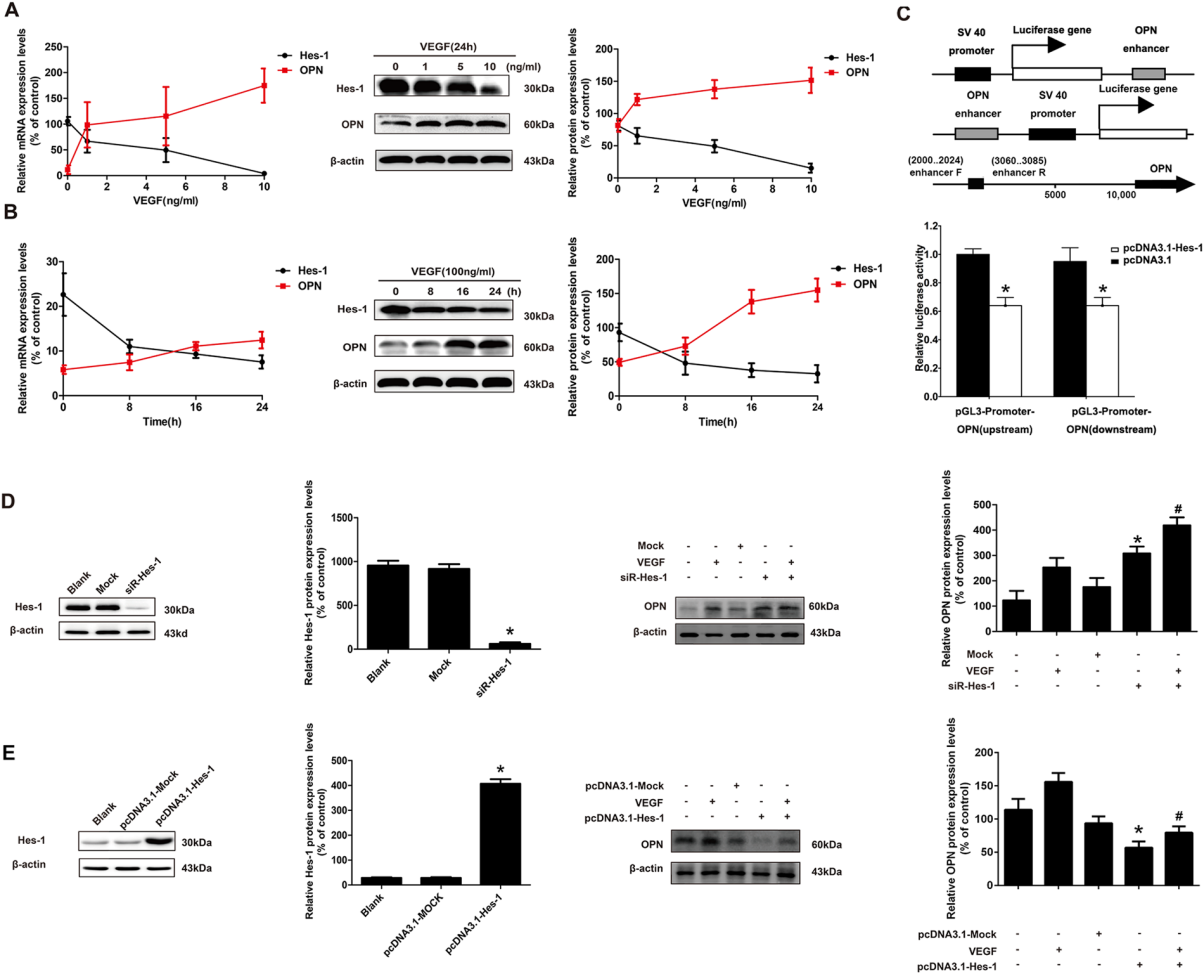
Hes-1 is involved in VEGF-induced up-regulation of OPN expression in HUVECs. (**A** to **B**) HUVECs were treated with VEGF at 0, 1, 5, and 10 ng/mL for 24 hours or treated with 10 ng/mL of VEGF for 0, 8, 16, and 24 hours, respectively. Hes-1 and OPN transcript levels were measured by real-time quantitative PCR and protein expression was assessed by western blotting. (**C**) Diagrammatic representation of the constructs used in the luciferase reporter assays and the luciferase activities of enhancer-SV40 promoter-Luc and SV40 promoter-Luc-enhancer compared with their counterparts. 293 T cells were cotransfected with pcDNA3.1-Hes-1 or a control vector and a luciferase reporter construct containing the the OPN enhancer. Luciferase activity was assayed 24 h after transfection. Firefly luciferase activity of each sample was normalized by Renilla luciferase activity. Data were normalized to the luciferase activity detected in cells transfected with the control vector. *p < 0.05 (**D**) Western blotting analysis of relative Hes-1 expression levels in HUVECs transfected with mock or siRNA-Hes-1 for 24 h. HUVECs were treated with mock or siRNA-Hes-1 and then incubated with or without 10 μg/mL of VEGF for 24 h. (**E**) Western blotting analysis of the relative Hes-1 expression levels in HUVECs transfected with pcDNA3.1-mock or pcDNA3.1-Hes-1 for 24 h. HUVECs were treated with pcDNA3.1-mock or pcDNA3.1-Hes-1 and then incubated with or without 10 μg/mL of VEGF for 24 h. OPN protein contents were measured by western blotting analysis. β-actin was used as a loading control. All results are presented as the mean ± SD of three independent experiments, each performed in triplicate. *p < 0.05 compared with the blank group. ^#^ *p* < 0.05, compared with the VEGF group.

Since this investigation revealed that Hes-1 was downregulated in atherosclerotic plaques, the regulation of Hes-1 during atherosclerosis formation and development was further investigated. To test this hypothesis, the role VEGF in the induction of the pathogenesis of atherosclerosis was investigated^25^. To determine whether Hes-1 was regulated by VEGF in HUVECs, real-time quantitative PCR and western blotting analyses were performed to detect Hes-1 mRNA and protein levels, respectively, in VEGF-treated HUVECs. The results revealed that VEGF inhibited Hes-1 expression at both the mRNA and protein levels in a dose-dependent manner (Fig. 3A). The inhibitory effects of VEGF on Hes-1 expression were gradually enhanced in a time-dependent manner, with the greatest effect observed at 24 h after VEGF treatment (Fig. 3B). These results indicated that the downregulation of Hes-1 observed in atherosclerotic plaques was probably induced by VEGF.

### Hes-1 suppressed VEGF-induced angiogenesis *in vitro* and *in vivo*

**Figure 4:**
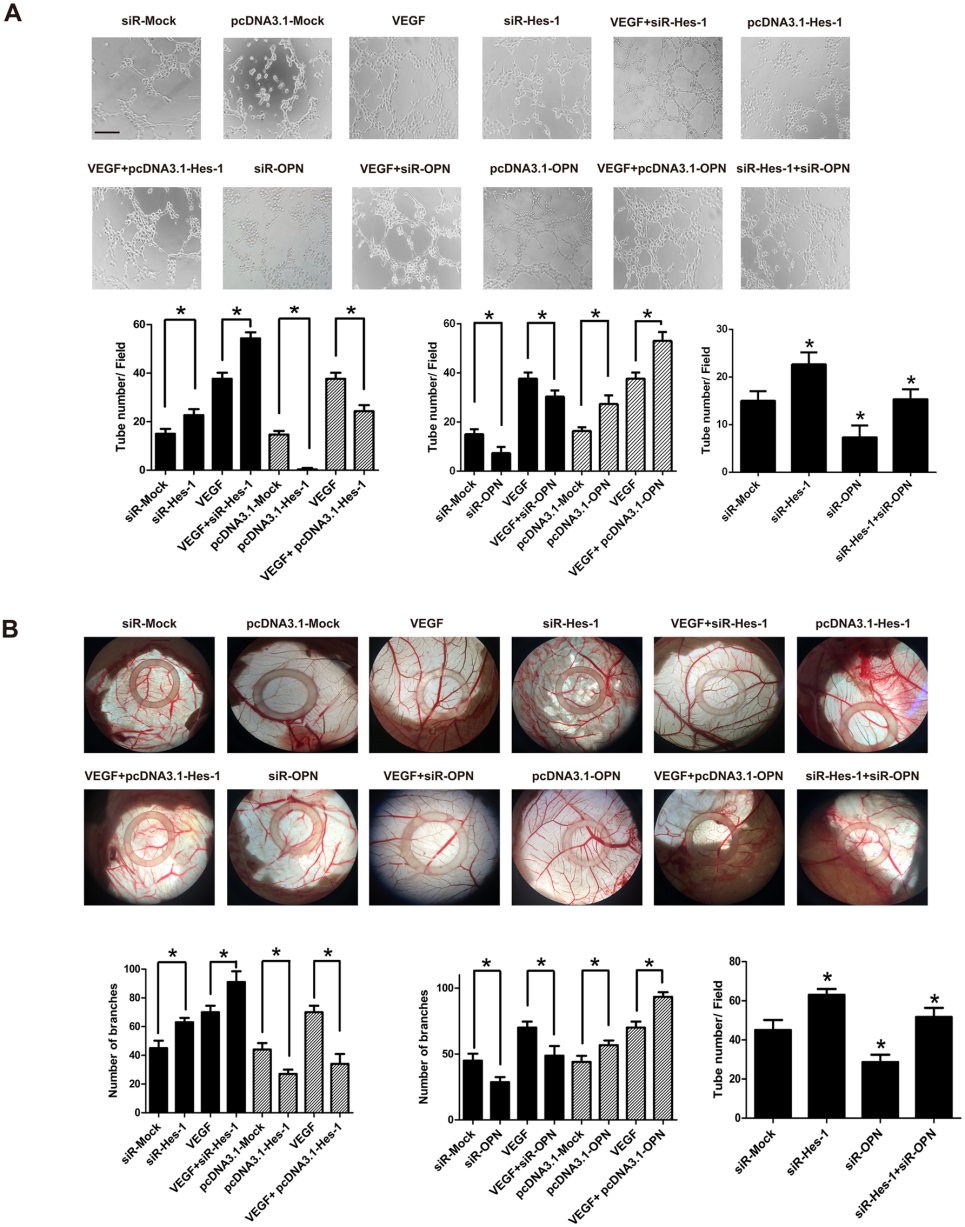
Hes-1 suppresses VEGF-induced angiogenesis via downregulation of OPN expression *in vitro* and *in vivo*. (**A**) HUVECs (1 × 10^5^) treated with siRNA/ pcDNA3.1of Hes-1 or OPN and then incubated with or without 10 ng/mL of VEGF for 24 h were seeded on matrigel-coated plates. After 6 h of incubation, endothelial cell tubular structure formation was photographed and analyzed. The number of tubular-like structures was counted and indicated as the number of branches per field. Averages of tubules were calculated from four fields in each sample. Pictures were taken at 20x magnification. *p < 0.05. Bar: 400 μm. (**B**) The CAM assay was done to assess angiogenesis *in vivo*, HUVECs (1 × 10^5^) treated with siRNA/ pcDNA3.1 of Hes-1 or OPN and then incubated with or without 10 ng/mL of VEGF for 24 h were pipeted directly into the center of the silicone ring resting on the CAM. Sealed the window and incubated the egg for 48 h to observe the result of neovascularization. The CAM surface was photographed at the same position with a digital camera, and the area of newly formed vessels was calculated. The data are presented as mean ± SD of three independent experiments. *p < 0.05.

VEGF is a well-known potent growth factor of endothelial cells and inducer of angiogenesis^17^. Several recent studies reported that Hes-1 is essential for angiogenesis in various types of cancer^26^. Kitagawa *et al*.^27^ also demonstrated that Hes-1 regulated vascular remodeling and the arterial fate of ECs in brain development, indicating an important role of Hes-1 in angiogenesis regulation. Since the findings of this investigation revealed that Hes-1 was downregulated in atherosclerotic plaques and VEGF-treated HUVECs, the role of Hes-1 in VEGF-induced angiogenesis was further investigated using the tube formation test. First, the capability of VEGF to induce angiogenesis, as indicated by tube formation of HUVECs was verified. As shown in Fig. 4A, although HUVECs were able to form vessel tubes in Matrigel, cells treated with VEGF formed a greater abundance of tubes. After Hes-1 knockdown, HUVECs formed more vessel tubes in Matrigel than cells transfected with control siRNA, suggesting Hes-1 knockdown *per se* could improve the basal angiogenic capability of HUVECs. Following VEGF induction, Hes-1 knockdown was found to significantly increase the number of vessel tubes, as compared to cells transfected with control siRNA, indicating that Hes-1 knockdown enhanced VEGF-induced angiogenesis. Next, the ability of Hes-1 overexpression to suppress VEGF-induced angiogenesis was investigated, which showed that Hes-1 overexpression significantly reduced the basal angiogenic capability of HUVECs and markedly suppressed the enhancement of VEGF-induced angiogenesis, indicating that Hes-1 overexpression indeed suppressed VEGF-induced angiogenesis. Similar to the *in vitro* studies, as a positive control *in vivo*, VEGF significantly induced angiogenesis on chick chorioallantoic membrane. Compared to this control, there was a high level of number of branches in Hes-1 knockdown cells and it was significantly augmented on chorioallantoic membrane following VEGF induction, indicating that Hes-1 knockdown enhanced new branched blood vessels formation. Reversely, VEGF-induced angiogenesis was also suppressed by Hes-1 overexpression (Fig. 4B). Taken together, these data revealed that Hes-1 is a negative regulator of VEGF-induced angiogenesis.

### Up-regulation of OPN in human atherosclerotic plaques

OPN in human atherosclerotic plaques is reportedly closely associated with the severity of atherosclerosis^28–31^. More importantly, OPN is recognized as a critical angiogenic factor in the regulation of proliferation and migration of vascular endothelial cells associated with neointima formation^32, 33^. In addition, Matsue *et al*.^17^ showed that Hes-1 is a potential regulator of OPN. As indicated by the microarray results, OPN expression was increased by 7.02-fold (*p* < 0.005) in atherosclerotic plaques. To further investigate the role of OPN upregulation in atherosclerotic plaques, OPN expression was determined in four atherosclerotic plaques and four normal arterial intima tissues using real-time PCR, immunohistochemical and immunofluorescence. Real-time PCR analysis showed that OPN mRNA levels were significantly higher in atherosclerotic plaques than in normal arterial intima tissues (Fig. 1B). Immunohistochemical staining for OPN was demonstrated within the arterial intima. The mean optical density of OPN staining was significantly greater in atherosclerotic plaques and revealed a high expression level of OPN (Fig. 2A). CD31 and OPN double immunofluorescence labeling showed that OPN was expressed in the ECs of neovessels in atherosclerotic plaques. Immunofluorescence results also showed a significant elevation in OPN expression in atherosclerotic plaques, as compared to normal arterial intima (Fig. 2B). These results further confirmed that OPN was upregulated in ECs of neovessels in atherosclerotic plaques.

### VEGF upregulated OPN expression in HUVECs

Since this investigation revealed that Hes-1 was downregulated, while OPN was upregulated, in atherosclerotic plaques, it was hypothesized that OPN was also regulated by Hes-1 during atherosclerosis formation and development. To examine if OPN was regulated by VEGF in HUVECs, real-time quantitative PCR and western blotting analyses were performed to detect OPN mRNA and protein levels, respectively, in VEGF-treated HUVECs. The results revealed that VEGF promoted OPN expression at both the mRNA and protein levels in a dose-dependent manner (Fig. 3A). The promoting effects of VEGF on OPN expression were gradually enhanced in a time-dependent manner, with the greatest effect observed at 24 h after VEGF treatment (Fig. 3B). These results indicated that the OPN upregulation observed in atherosclerotic plaques was probably regulated by VEGF.

### Hes-1 regulated VEGF-induced upregulation by regulating OPN in HUVECs

Hes-1 overexpression was recently shown to reduce OPN mRNA levels in osteoblastic cells, suggesting that Hes-1 is a potential regulator of OPN^15^. Since the results of this study revealed that Hes-1 was downregulated and OPN was upregulated in atherosclerotic plaques *in vivo*, and that VEGF downregulated Hes-1 and upregulated OPN in HUVECs *in vitro*, we hypothesized that Hes-1, as a transcriptional factor, was the key mediator in VEGF-induced OPN upregulation. To examine the effect of Hes-1 on the promoter activity of OPN, we generated the pGL3-OPN plasmid, an OPN promoter luciferase reporter. Additionally, transient transfection was performed in 293 T cell models using the pGL3-OPN plasmid. We then examined the effect of Hes-1 on the promoter activity of OPN using pGL3-OPN in luciferase reporter gene assays. Our data showed that the luciferase activities of enhancer-SV40 promoter-Luc and SV40 promoter-Luc-enhancer were dramatically reduced as compared with their counterparts. This may be attributable to the enhancer of the OPN have transcriptional suppression functions by Hes-1 (Fig. 3C).

Then the effect of knockdown and overexpression of Hes-1 on VEGF-stimulated OPN expression were investigated. Hes-1 in HUVECs was knocked-down by siRNA transfection. As shown in Fig. 3D, both protein and mRNA levels of Hes-1 were decreased by 50% in HUVECs after siRNA transfection. Immunoblot analysis showed that Hes-1 knockdown significantly upregulated OPN without VEGF induction and significantly enhanced OPN upregulation after VEGF induction. Next, the effects of transfection with recombinant plasmid over-expressing Hes-1 (pcDNA-Hes-1) on protein levels of OPN were examined. As shown in Fig. 3E, HUVECs transfected with pcDNA-Hes-1 successfully overexpressed Hes-1 at the protein level and overexpressing Hes-1 downregulated basal levels of OPN. After VEGF treatment, the elevation in OPN protein levels by VEGF was markedly compensated by Hes-1 overexpression. These data demonstrated that Hes-1 was a transcriptional mediator of VEGF-regulated OPN expression and that VEGF upregulated OPN partially through the downregulation of Hes-1, indicating that decreased Hes-1 expression contributed to the increase in OPN expression in VEGF-treated HUVECs and likely in atherosclerotic plaques.

### Hes-1 regulated VEGF-induced angiogenesis by regulating OPN *in vitro* and *in vivo*

Since OPN has been confirmed as a critical angiogenic factor^34^ and Hes-1 might regulate VEGF-induced angiogenesis through OPN regulation, then we validated the role of OPN in VEGF-induced angiogenesis using tube formation test and CAM assay. As shown in Fig. 4A and B, HUVECs formed less vessel tubes and branched blood vessels with OPN knockdown than control. Following VEGF induction, OPN knockdown was found to markedly suppress VEGF-induced angiogenesis. Then, overexpression of OPN was showed to promote VEGF-induced angiogenesis. Moreover, transfection of Hes-1 siRNA nearly rescued the suppression of angiogenesis by OPN knockdown. Taken together, these data revealed Hes-1 as a negative regulator of VEGF-induced angiogenesis through regulating OPN.

## Discussion

Angiogenesis is an important process in the progression of atherosclerotic plaque formation^35^. However, the underlying mechanism of angiogenesis regulation in atherosclerotic plaque formation remains unclear. The results of the present study demonstrated that Hes-1 expression was significantly decreased, while that of OPN was increased, in atherosclerotic plaques (Fig. 1). Furthermore, both Hes-1 and OPN were expressed in ECs of neovessels in atherosclerotic plaques, and expression of VEGF, a key agiogenic inducer, downregulated Hes-1 expression and upregulated that of OPN in HUVECs (Fig. 2). Specifically, we demonstrated that Hes-1 act as a transcriptional mediator which inhibited OPN levels (Fig. 3C). More importantly, Hes-1 knockdown in HUVECs enhanced VEGF-induced OPN upregulation and promoted angiogenesis, but Hes-1 overexpression suppressed VEGF-induced OPN upregulation and inhibited angiogenesis (Figs 3 and 4), indicating that increased OPN expression in the ECs of atherosclerotic plaques was caused by the decrease in Hes-1 expression. Since OPN was shown to promote VEGF-induced angiogenesis, the decreased expression of Hes-1 in the ECs of neovessels in atherosclerotic plaques might have promoted angiogenesis by upregulating OPN. Hence, restoring expression of Hes-1 may be a promising therapeutic modality to prevent formation of vulnerable plaques.

The results of the present study showed the Hes-1 expression was significantly decreased, while that of OPN was increased, in atherosclerotic plaques. Although the mechanisms underlying regulation of Hes-1 and OPN remain unclear, these *in vitro* data demonstrated that VEGF administration downregulated Hes-1 expression and upregulated OPN expression, indicating that dysregulation of Hes-1 and OPN in the ECs of neovessels in atherosclerotic plaques was likely induced by VEGF.

VEGF is well recognized as a key factor required for the development of atherosclerosis and is a potent growth factor of ECs and a critical inducer of angiogenesis that has been correlated to the progression and vulnerability of atherosclerotic plaques^36, 37^. Recent studies have uncovered hypoxia-inducible factor 1 (HIF-1) as well as HIF-2, a gene highly homologous to HIF-1, bind to specific enhancer elements, resulting in increased VEGF gene transcription^38^. Importantly, other studies have implicated the PI3 kinase/Akt pathway in the regulation of HIF-mediated responses in a hypoxia- independent manner^39^. Several major growth factors, including epidermal growth factor, TGF-α, TGF-β, keratinocyte growth factor, IGF-I, FGF, and PDGF, and hormones are also important regulators of VEGF gene expression^40^. Additionally, Amano *et al*. have shown that PGE2-EP3 receptor signaling also plays a significant role in up-regulating VEGF in stromal cells^41^. Takeshita *et al*. demonstrated that Notch signaling acts downstream of VEGF signaling and was critical for VEGF-induced postnatal angiogenesis^26^. Hes-1 has been shown to be a target gene of the Notch intracellular domain, which is thought to translocate the nucleus to activate Hex-1 transcription^42^. According to these findings, it is possible that VEGF regulates Hes-1 through Notch signaling. However, there are six Notch ligands (Dll1–4, Jagged1, and Jagged2) and six receptors (Notch 1–6)^43^. Thus, the existence of such multiple components at each step of Notch signaling leads one to speculate that different ligands might be linked to distinct receptors and effectors^7^. Interestingly, Qi *et al*. showed that interacting between Hes-1 and Runx2 enhance basal and 1, 25(OH)2D3-induced OPN transcription in osteoblastic cells^44^, and Jung *et al*. demonstrated that Hes1 stimulates Runx2 activity in osteoblast differentiation^45^. However, according to Garg *et al*., Notch1 and Hes-1 repressed the activity of Runx2 in COS7 cells^46^. Additionally, Notch-Hes pathway was demonstrated to negatively regulate bone marrow mesenchymal stromal cells osteogenesis through inhibition of Runx2 transcriptional activity^47^. Importantly, Hes-1 is antagonizing the expression or function of a wide range of B-class bHLH transcriptional activators, including for example, Math1 in intestine and Ngn3 in pancreas^48^. In accordance with the findings of the present study, silencing Hes-1 enhanced the VEGF-induced upregulation of OPN, while inhibiting the overexpression of Hes-1 in HUVECs (Fig. 3). Although the mechanism underlying Hes-1 regulation of OPN remains undetermined, the findings of this investigation suggest that VEGF upregulated OPN partially through the downregulation of Hes-1 and the upregulation of OPN in ECs of neovessels in atherosclerotic plaques was likely mediated by Hes-1. On the other hand, we postulate that the positively regulation of Hes-1 towards OPN reported in some studies maybe cell-type dependent or other reasons needing further investigation.

Hes-1, which is transcriptionally activated by Notch signaling, acts as a transcriptional repressor that plays an important role in developmental processes^7^. Some recent studies reported that Hes-1 is essential for angiogenesis in various types of cancer^26^. Moreover, Kitagawa *et al*. demonstrated that Hes-1 regulated vascular remodeling and arterial fate specification of ECs during brain development^27^. In HUVECs, delta-like 4/28-525 was reported to inhibit EC proliferation *in vitro* through by the induction of Hes-1 in a γ-secretase-dependent fashion and is responsible for the decrease in vascular sprouting observed in aortic rings from sDLL4/28-525-stimulated macrophages by incubation in conditioned media^49^. The results of the present study also showed that Hes-1 knock-down enhanced angiogenesis, while Hes-1 overexpression inhibited angiogenesis with or without VEGF induction, indicating a negative role of Hes-1 in angiogenesis. Although the regulatory role of Hes-1 is angiogenesis is not yet clear, we now provide evidence that OPN is regulated by Hes-1 during VEGF-induced angiogenesis. Since OPN is reportedly not only a cell attachment protein but also a cytokine, which delivers signals via a number of receptors including several integrins and CD44 in cells^10^. As a signaling molecule, OPN can modify gene expression and promote the migration of monocytes/macrophages upon an OPN gradient^50^, and also link to the proliferation and migration of vascular cells associated with neointima formation^32, 33^. Although we did not identify the signaling pathways mediated by OPN, several studies have shown that OPN plays a role in angiogenesis via different signaling pathways^13, 14^. According to Dai *et al*.^34^ OPN enhances angiogenesis directly through PI3K/AKT- and ERK-mediated pathways with VEGF acting as a positive feedback signal, and blocking the signal of OPN completely inhibited HUVEC motility, proliferation, and tube formation. Taken together, these findings indicate that Hes-1 modulates VEGF-induced angiogenesis through OPN regulation. In addition, Hes-1 has also been shown to regulate proliferation of certain cell types probably by cell cycle regulation^51^. It is also reported that Hes-1 evades differentiation and irreversible cell cycle arrest to maintain the reversibility of quiescence by controlling the choice between different out-of-cycle states^52^. Hence, it is also possible that Hes-1 modulates angiogenesis through regulation of EC proliferation in an OPN-independent manner.

Atherosclerosis is defined as a chronic inflammatory disease of the arterial wall arising from dysregulation of lipid metabolism and a maladaptive inflammatory response^53^. VEGF is also known to play a central role in inflammation and wound healing by controlling both angiogenesis and vascular permeability^54^, and OPN is recognized as a significant participant in the atherosclerotic inflammatory milieu^28^. Many studies have shown that OPN mRNA expression in human atherosclerotic plaques was closely associated with the severity of atherosclerosis^28–31^. Beyond its role in the regulation of intraplaque angiogenesis, previous works also showed that OPN plays an important role in inflammation regulation, such as the migration of macrophages^55^. Although less is known about the role of Hes-1 in the immune response and atherosclerosis, as compared to VEGF and OPN, the results of the present study identified Hes-1 as a novel regulator in atherosclerosis, especially intraplaque angiogenesis. However, the role of Hes-1 in inflammation requires further investigation.

## Conclusion

**Figure 5:**
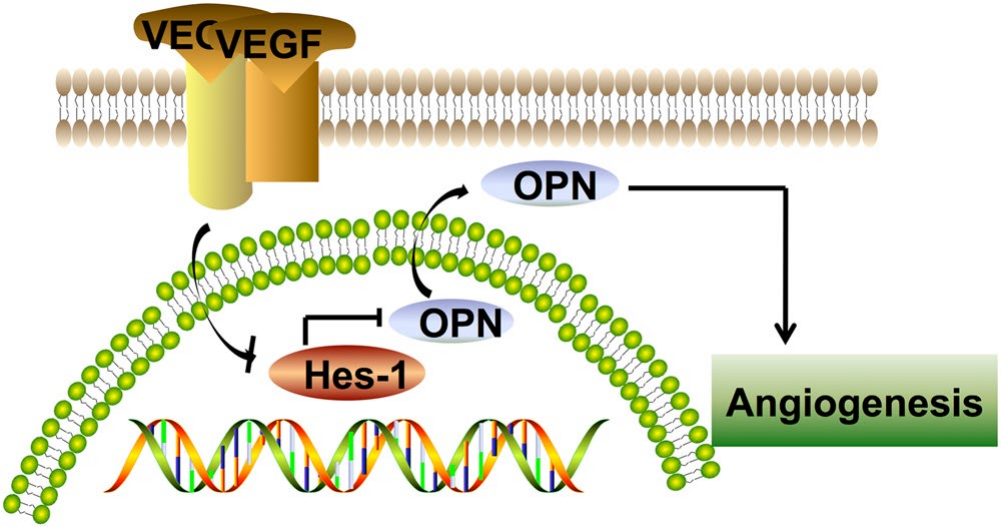
Schematic presentation that highlights the inhibitory effects of Hes-1 on VEGF-induced angiogenesis by targeting OPN gene in atherosclerotic plaques. The results of the present study revealed the following possible mechanism: Hes-1 acted as a transcriptional mediator which inhibited OPN levels. Hes-1 suppressed VEGF-induced angiogenesis via downregulation of OPN expression.

As displayed in Fig. 5, our study revealed for the first time that Hes-1 acted as a transcriptional mediator which inhibited OPN levels. This is the first study to reveal that Hes-1 in ECs plays an important role in angiogenesis by OPN regulation, and that Hes-1 downregulation and OPN upregulation may contribute to the progression and vulnerability of atherosclerotic plaques. This knowledge may shed new light on the therapy of vulnerable plaque formation in patients with atherosclerosis.

## Materials and Methods

### Ethics and consent

Informed consent was obtained from each patient and ethical approval was obtained from the Institutional Review Board of Nanfang Hospital of Southern Medical University. Informed consent was obtained from all subjects. All experiments were performed in accordance with relevant guidelines and regulations. None of the patients received medication before surgery. Clinical and pathological data, including pathological grading and tissue origins, are listed in Table 1.

### Sources of tissue samples

The specimens were collected between April 2015 and June 2015 in the Department of Vascular Surgery, Nanfang Hospital, Southern Medical University. Four samples of renal arteries with atherosclerotic plaques and four samples of renal arteries free of atherosclerosis were obtained during renal transplantation surgery. Tissues were collected and fixed with formalin or frozen at −80 °C. Fixed tissues were used for immunohistochemical and immunofluorescent studies. Fresh, unfixed material was used for protein and RNA extraction.

### Cell Culture

HUVECs were purchased from Lonza (Walkersville MD, USA), cultured in Dulbecco’s modified Eagle’s medium (Gibco®; Life Technologies, Carlsbad, CA, USA) supplemented with 10% fetal bovine serum (Gibco®) under standard culture conditions (5% CO_2_, 37 °C), and seeded in 6- or 96-well plates, or 60-mm dishes. Recombinant VEGF was purchased from Sigma Chemical Company (St. Louis, MO).

### Microarray analysis

Briefly, total RNA was isolated from each artery sample. RNA integrity was assessed using standard denaturing agarose gel electrophoresis and quantified using a NanoDrop ND-1000 spectrophotometer (Thermo Fisher Scientific, Waltham, MA, USA). The Agilent array platform was employed for microarray analysis (Agilent Technologies, Inc., Santa Clara, CA, USA). Sample preparation and microarray hybridization were performed using the manufacturer’s standard protocols with minor modifications. Briefly, mRNA was purified from 1 μg of total RNA after removal of rRNA using the mRNA-ONLY™ Eukaryotic mRNA Isolation Kit (Epicentre Biotechnologies, Madison, WI, USA). Then, each sample was amplified and transcribed into fluorescent cRNA along the entire length of the transcripts without 3′ bias utilizing a random priming method. The labeled cRNAs were hybridized onto the Human mRNA 3.0 (8 × 60 K; Arraystar, Inc., Rockville, MD, USA). After the slides were washed, the arrays were scanned using the Agilent Scanner G2505B. Agilent Feature Extraction software (version 11.0.1.1) was used to analyze acquired array images. Quantile normalization and subsequent data processing were performed using the GeneSpring GX v11.5.1 software package. After quantile normalization of the raw mRNA data, at least three of six samples that were flagged as “Present” or “Marginal” (“All Targets Value”) were chosen for further data analysis. Differentially expressed mRNAs to statistically significant levels were identified through Volcano Plot filtering. Pathway analysis was applied to determine the roles of differentially expressed mRNAs in various biological pathways. Finally, hierarchical clustering was performed to identify distinguishable mRNAs expression patterns among the samples.

### Immunohistochemical analysis

Arteries from patients were formalin-fixed, mounted on end, and wax-embedded. Sections were rehydrated and stained by the immunoperoxidase technique using antibodies against Hes-1 (diluted 1:100; Abcam plc, Cambridge, MA, USA), OPN (1:100, Abcam plc), and CD31 (1:100; Abcam plc)^24^. For this procedure, each artery specimen was sectioned to thicknesses of 5 μm to assess staining intensity at the same time to minimize differences in staining conditions. Staining was quantified by optical density^56^. Briefly, computerized images of each section were captured using a digital camera mounted on an Olympus BX51 microscope (Olympus Corporation, Tokyo, Japan) and assessed using Optimis software (Optimis Systems Limited, Epsom, New Zealand). Three areas of maximal staining were identified and the mean optical density of these sites was measured using Scion software (Scion Software Solutions, Hyderabad, Telangana, India). The optical densities were averaged and background measurements from serial sections in which the primary antibody was omitted from the resultant values. The reproducibility of the optical density measurements was assessed in eight specimens examined on two separate occasions 7 days apart by the same observer.

### Double immunofluorescence staining

Each artery section was subjected to double immunofluorescence staining of CD31 and Hes-1/ OPN. Briefly, deparaffinized sections were incubated with anti-CD31 antibody for 1 h at room temperature and then incubated with secondary antibodies conjugated to alkaline phosphatase-labeled polymers using the HISTOFINE immunohistochemical staining system (Nichirei Biosciences Inc., Tokyo, Japan). Color development was performed using the Vector Red alkaline phosphatase substrate kit (Vector Laboratories, Inc., Burlingame, CA, USA). Antigen retrieval was performed by incubating the sections with 20 mg/ml of proteinase K for 6 min at room temperature and microwaving in 10 mmol/L of citrate buffer (pH 6.0) for 10 min. Then, the sections were incubated overnight at 4 °C with anti-Hes-1 antibody (diluted 1:100) and anti-OPN antibody (diluted 1:100). Alexa Fluor 488 (10 μg/ml; Molecular Probes™, Thermo Fisher Scientific, Eugene, OR, USA) was used as a secondary antibody. Nuclei were stained with 4′6-diamidino-2-phenylindole and the sections were observed under an immunofluorescence confocal microscope.

### Luciferase reporter gene assays

To construct the luciferase reporter vector for the OPN enhancer, a 1045 bp fragment derived from the region of human OPN (GenBank accession NC_000004) was amplified by PCR from the human genomic DNA. The PCR product was then purified and subcloned into the SacI/ XhoI sites and BamHI/ SaII of the pGL3-Promoter vector, to generate the enhancer-SV40 promoter-Luc and SV40 promoter-Luc-enhancer construct. The CDS of Hes1 were generated by PCR with corresponding primers and cDNAs. The resulting amplicons were subcloned into the HindIII and XhoI sites of the pcDNA3.1 vector (Invitrogen). 293 T cells were grown in monolayers in 24-well plates and the cells in each well were transfected with 0.4 μg of the reporter construct according to the manufacturer’s instructions. After 24 h, the luciferase activities were measured with the Dual Luciferase Reporter Assay System (Promega) on a Turner Designs TD-20/20n luminometer (Promega). The amount of firefly luciferase activity was normalized to the amount of Renilla luciferase activity. The results shown are representative of at least three independent experiments (each performed in triplicate).

### RNA isolation and real-time quantitative PCR analysis

Total RNA from tissues and cells was extracted using TRIzol reagent (Invitrogen Corporation, Carlsbad, CA, USA), according to the manufacturer’s instructions, and mRNA levels were determined by real-time quantitative PCR using an ABI 7500 Fast Real-Time PCR system (Applied Biosystems, Foster City, CA, USA) with SYBR-Green Detection chemistry (Takara Bio, Inc., Shiga, Japan). All samples were measured in triplicate and the mean value was considered for comparative analysis. Quantitative measurements were determined using the 2^−ΔΔCT^ method with glyceraldehyde 3-phosphate dehydrogenase (GAPDH) as an internal control.

### Western blotting analysis

Proteins were extracted from cultured cells using radioimmunoprecipitation assay buffer (Biocolor Ltd., Belfast, Northern Ireland, UK) and protein concentrations were quantified using a bicinchoninic acid protein assay kit (Nanjing KeyGen Biotech. Co. Ltd., Nanjing, China). Denatured samples were separated by 10% sodium dodecyl sulfate-polyacrylamide gel electrophoresis and subjected to western blotting analyses using rabbit polyclonal anti-Hes-1 antibodies, rabbit polyclonal anti-OPN antibodies, and rabbit polyclonal β-actin-specific antibodies (all from Abcam plc). The proteins were visualized using an ECL Plus Western Blot Detection System (Amersham Biosciences, San Diego, CA, USA).

### Transfection with small interfering RNA (siRNA)

 SiRNAs against Hes-1 and an irrelevant 21-nucleotide control siRNA (negative control) were purchased from Souzhou Ribo Life Science Co., Ltd. (Guangdong, China). Cells (2 × 10^6^ cells/well) were transfected using Lipofectamine 3000 transfection reagent (Thermo Fisher Scientific) for 24 h, according to the manufacturer’s instructions. After 24 h of transfection, western blotting was performed to verify knockdown efficiency.

### Construction of recombinant plasmids

The PIRES2-EGFP and PCR-XL-TOPO vectors (containing Hes-1, which was assembled with chemically synthesized oligos through PCR) were purchased from Invitrogen Corporation. The fragment of *Eco*RI-Hes-1-IRESEGFP-*Xho*I was amplified using the templates of the PCR-XL-TOPO and PIRES2-EGFP vectors, respectively. *Eco*RI-Hes-1-IRES-EGFP-*Xho*I was joined by the two above-mentioned segments using overlap PCR. Gel electrophoresis was performed and the relevant band was excised from the gel, double enzyme-digested with *Eco*RI/*Xho*I, incorporated into the pcDNA3.1(+) vector, and then transformed into competent *Escherichia coli* DH5a cells for further amplification and use. The recombinant pcDNA3.1-Hes-1 plasmids were verified by sequencing and transfected using Lipofectamine 3000 transfection reagent, according to the manufacturer’s instructions. Stable transformants were selected for 4 weeks and isolated by a single cell manipulation technique.

### Tube formation test

The tube formation test is a well-established assay to detect the formation of three-dimensional vessels and to assess angiogenesis *in vitro* ^57^. Matrigel (Sigma-Aldrich Corporation, St. Louis, MO, USA) was prepared in a 96-well plate, according to the manufacturer’s instructions. HUVECs were seeded into the coated wells at a density of 1 × 10^5^ cells/well and incubated at 37 °C for full development of vessel-like tube structures. After 6 h, photographs were taken under a microscope (Nikon Corporation, Tokyo, Japan).

### Chick Embryo Chorioallantoic Membrane (CAM) Assay

Chick embryo chorioallantoic membrane (CAM) assay was carried out for *in vivo* angiogenesis assays as previously described, which is also the most widely used one to study angiogenesis^58^. After incubation for 6 days, a 1–2 cm^2^ square window was made on the air sac to expose the CAM window was opened at the blunt end of the eggs and the shell membrane was removed to expose the CAM. Briefly, a silicone ring 1 cm in diameter was applied to the CAM surface of embryos. Then, pipet cell directly into the center of the silicone ring resting on the CAM. Sealed the window and incubated the egg for 48 h to observe the result of neovascularization. The CAM surface was photographed at the same position with a digital camera, and the area of newly formed vessels was calculated.

### Statistical analysis

Data were analyzed using the Student’s *t*-test or one-way analysis of variance, followed by the Student–Newman–Keuls test using SPSS v20.0 statistical software (SPSS, Inc. Chicago, IL, USA) and the results are expressed as the mean ± standard deviation (SD). A two-tailed probability (*p*) value of <0.05 was considered statistically significant.
